# PHYSICAL ACTIVITY ATTENUATES THE INFLUENCE OF ALZHEIMER’S BIOMARKERS ON COGNITIVE FUNCTION

**DOI:** 10.1093/geroni/igae098.1197

**Published:** 2024-12-31

**Authors:** Ryan Dougherty, Anja Soldan, Corinne Pettigrew, Barry Greenberg, Adam Spira, Abhay Moghekar, Marilyn Albert

**Affiliations:** Rutgers University, Stoughton, Wisconsin, United States; Johns Hopkins University, Baltimore, Maryland, United States; Johns Hopkins University School of Medicine, Baltimore, Maryland, United States; Johns Hopkins University, Baltimore, Maryland, United States; Johns Hopkins University, Baltimore, Maryland, United States; Johns Hopkins University School of Medicine, Baltimore, Maryland, United States; Johns Hopkins University School of Medicine, Baltimore, Maryland, United States

## Abstract

Alzheimer’s disease (AD) is characterized by the abnormal accumulation of amyloid-β (plaques), and tau (neurofibrillary tangles) that can be quantified in vivo through cerebrospinal fluid (CSF) sampling. Physical activity has emerged as a possible modifier of AD risk; however, the influence of physical activity on CSF biomarkers and cognitive function remains unclear. We examined whether higher levels of physical activity favorably modify associations between AD CSF biomarkers and cognitive function. One hundred and seventeen adults free of dementia from the BIOCARD study (mean age 72.2±8.0 years, 70% women) wore a wrist accelerometer for one week, underwent lumbar puncture to collect CSF, and completed a comprehensive neuropsychological exam. Covariate-adjusted linear regression analyses were used to examine whether physical activity (total activity counts over the 10 most active hours of the day) moderates the association between AD CSF biomarkers [amyloid-β (Aβ42/40), total tau, phosphorylated tau (p-tau181)] and cognitive composite scores (episodic memory, executive function). There were significant interactions between physical activity and total tau (p=.004) as well as physical activity and p-tau181 (p=.016) for the executive function composite score. Among those with elevated total tau or p-tau181, participants with greater levels of physical activity exhibited significantly higher executive function composite scores compared to participants with lower levels of physical activity. In contrast, there were no significant interactions for episodic memory, and physical activity did not interact with Aβ42/40 (all interactions p>.05). These findings suggest that a physically active lifestyle may play a protective role against cognitive decline through tau pathways.

